# Prevalence of Urinary Schistosomiasis in Migrants in Apulia, a Region of Southern Italy, in the Years 2006–2016

**DOI:** 10.1155/2017/8257310

**Published:** 2017-12-25

**Authors:** Gaetano Brindicci, Carmen Rita Santoro, Vittoriana De Laurentiis, Carmen Capolongo, Maria Elena Solarino, Roberta Papagni, Emanuela Ciracì, Pietro Gatti, Daniela Loconsole, Rosa Monno, Laura Monno, Giuseppe Miragliotta, Gioacchino Angarano

**Affiliations:** ^1^Clinic of Infectious Diseases, Hospital-University Polyclinic, University of Bari, Bari, Italy; ^2^Operative Unit of Microbiology and Virology, Hospital-University Polyclinic, University of Bari, Bari, Italy; ^3^Operative Unit of Radiodiagnostic, Di Venere Hospital, Bari, Italy; ^4^Internal Medicine Unit, Ostuni Hospital, Ostuni, Italy; ^5^Department of Biomedical Science and Human Oncology, Hospital-University Polyclinic, University of Bari, Bari, Italy

## Abstract

Schistosomiasis is the most prevalent tropical disease in the world after malaria. According to the World Health Organization, the disease afflicts more than 240 million people in about 80 countries. Recently, an epidemiological surveillance study performed between 1997 and 2010 by the European Network for Tropical Medicine and Health Travel regarding schistosomiasis between immigrants and travelers has been published. No data are available in the literature regarding the situation in South Italy. Herein, we report the prevalence of urinary schistosomiasis in a population of migrants in Apulia referring to our outpatient clinic for immigrant diseases in the period 2006–2016. Since all cases of schistosomiasis were related to the last three years of observation, the demographic and clinical characteristics of the study population were compared before and after 2014. Nearly 51% of all patients visited (1762) were from high/moderate endemic countries for schistosomiasis, and nine cases of urinary schistosomiasis were diagnosed. Prevalence was 1% among migrants from endemic areas and 10% in those from Mali and Senegal. Our findings confirm that schistosomiasis is a widespread infection among immigrants, even if it is often underdiagnosed because of the multifaceted clinical presentation. Changes in migratory dynamics can affect clinical observations very quickly.

## 1. Introduction

Schistosomiasis is a parasitic disease caused by trematodes of the genus* Schistosoma*, described for the first time in 1851 by Theodor Bilharz [1]. After malaria, schistosomiasis remains the most prevalent tropical disease in the world [2, 3]. The World Health Organization (WHO) reported more than 240 million cases spread out largely in about 80 developing countries [2]. In humans, the disease can be caused by five different species of* Schistosoma*,* (S. haematobium, S. mansoni, S. japonicum, S. mekongi,* and* S. intercalatum)* that, in the cercariae life stage, can cross the intact skin of subjects as a consequence of a direct contact with water sources containing infectious cercariae [4].

*S. haematobium*, responsible for urinary schistosomiasis, is endemic in Africa, in the Arabian Peninsula, and in some countries of Middle East [5, 6]. High-burden countries, where the prevalence of schistosomiasis is ≥50%, are numerically limited and confined to a restricted area of Sub-Saharan Africa whereas countries to moderate endemicity (10–49% prevalence) include much of Sub-Saharan Africa and Yemen. According to WHO these countries are considered at high risk of transmission [7, 8].

Because of the increasingly large migratory flows from developing countries and of mass tourism, schistosomiasis might become a public health problem in the West; in the USA, 400,000 cases/year of Schistosomiasis are estimated, of which most occur in migrants from endemic areas (Puerto Rico, Brazil, Philippines, and Middle East) [9].

Recently a study in the metropolitan area of Barcelona confirmed a high prevalence of urinary schistosomiasis among migrants from Western Africa who have lived in Europe for many years [10].

According to the Eurostat data, in 2012 Italy was the third European country for its absolute number of foreign residents (4.8 million) [11] and although the majority of the migrant population (>60%) is concentrated in the North [12], in 2014 Apulia was the region with the highest increase of migrants.

Moreover a recent epidemiological surveillance study performed between 1997 and 2010 by the European Network for Tropical Medicine and Health Travel reported cases of schistosomiasis between immigrants and travelers. The network that included four Northern Italy centers (Verona, Milan, Udine, and Turin) advised an elevated prevalence of schistosomiasis among migrants from Western Africa [13]. An even greater prevalence of schistosomiasis has been reported by Beltrame et al. among migrants recently landed in Italy and resident in Veneto [14].

A multicentre cross-sectional study among immigrants accessing health care facilities in seven north central Italian cities (Verona, Brescia, Bologna, Florence, Modena, Parma, and Rome) reported a urinary schistosomiasis prevalence of less than 6% [15]. To our knowledge, no data are available in literature regarding the situation in South Italy.

Herein, we describe the prevalence of urinary schistosomiasis in a population of migrants in Apulia, the majority of whom arrived in Europe less than a year, who referred to the outpatient clinic for immigrant diseases c/o the Infectious Diseases Clinic of Bari over a period running from 2006 to 2016. In addition, since all cases of urinary schistosomiasis occurred in the period 2014–2016, we retrospectively analyzed demographic and clinical data on immigrants to assess the factors that could have led to this change in prevalence.

## 2. Patients and Methods

All consecutive immigrant individuals referring to our outpatient clinic for the first time between January 2006 and December 2016 were retrospectively included in the analysis.

Immigrants were mostly just arrived in Italy and all were with permit to stay, whether they were irregularly present on the country or they were awaiting for regularization. About a quarter were sex workers. Almost the entire sample accessed our services through the help of volunteering associations active in the area. To a lesser extent, they were sent to us by other health facilities when an infectious disease was suspected.

After screening (blood count, kidney and liver-function tests, serum electrolytes, protein electrophoresis, inflammatory markers, B and C hepatitis serology, HIV, syphilis, and urine examination), patients with eosinophilia and hematuria were offered second-level tests (urine and stool collection, assay serum for total IgE, serology to detect antibodies anti-*Trichinella spiralis*,* Toxocara canis*,* Schistosoma *spp., and* Echinococcus *spp.). All the patients underwent an ultrasound scan of the abdomen.

Confirmed schistosomiasis was defined by the presence of* S. haematobium* eggs revealed by microscopy either in urine or on biopsy. Otherwise, the diagnosis was defined as “probable” in the presence of positive serology performed by indirect hemagglutination technique (IHA), fluorescent antibody test (FAT) or enzyme-linked immunosorbent assay (ELISA), “suspect” in the presence of symptoms and signs, or radiological images or blood tests suggestive of urinary schistosomiasis or in the case of the disappearance of hematuria and clinical symptoms after treatment with praziquantel. A direct diagnosis was performed on three nonconsecutive urine samples collected between 10:00 am and 2:00 pm, when the elimination of eggs in the urine is the highest; furthermore, the patient was asked to collect urine after doing about 20 rapid knee-bends.

The sample was centrifuged at 2000 g for 2 minutes and the sediment was studied in the fresh microscopic examination at 10x to detect the presence of the typical eggs ranging in size between 62 and 150 *μ*m.

A commercial ELISA kit (Schistosoma Antibody Assay, Scimedx Corporation, Ali-Fax, Padova, Italy) was used for the detection of specific antibodies. According to the manufacturer's instruction, the test is considered positive for an absorbance value ≥ 0.2 OD units. The specificity and sensitivity of the test for the infection with Schistosoma spp. are 100% and 85%, respectively.

Ultrasonography was also performed with an ultrasound Esaote MyLab 50 basic version and 3–5 MHz convex probe. Patients were invited to present a full bladder, and the study of the bladder wall was carried out by both longitudinal and transversal scans before and after urination.

### 2.1. Statistical Analysis

Since all schistosomiasis cases in fact were diagnosed in period 2014–2016, the demographic and clinical characteristics (age, sex, prevalence of eosinophilia, and countries of origin) of this group of patients were compared to those of migrants visited during 2006–2013 to see if there was any difference that might have affected our results. The data were then processed using the software package STATA 12.0. *χ*^2^-test, which was used to compare proportions. A *t*-test was used to compare the means. If a *p* value was below 0.05, the difference between proportions was considered to be statistically significant.

## 3. Results

Between 2006 and 2016, 1762 immigrant patients were visited; patients were mostly males (65.8%) with an average age of 28 years. A 11.8% prevalence of eosinophilia (an absolute number of eosinophils > 400 cells/mm^3^) was recorded in the period 2006–2016. Urine examination was available for a limited number of cases. Nearly 51% of patients (896) came from high or moderate endemic countries for schistosomiasis, and in nine cases a diagnosis of urinary schistosomiasis was made.

Four patients had leukocyturia, and so many presented significant proteinuria. One patient reported a relevant suprapubic pain associated with dysuria and increased urinary frequency

**Figure 1:**
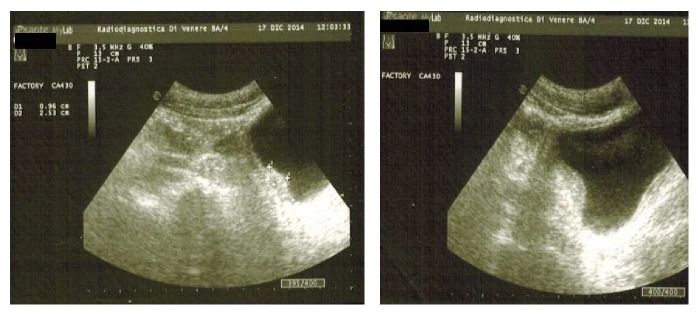
A hyperechoic intraluminal nonmobile vegetations of the bladder wall.

The relevant personal and clinical details of the nine patients are reported in Table 1. All the patients were males, with a mean age of 22.3 years (range 17–41 years: 5/7 from Mali and 2/7 from Senegal, a patient from Eritrea and the last one from Nigeria). All had been in Italy for less than one year, and only two had attended primary schools in their country of origin. Although everybody was affiliated to volunteer associations, five individuals were residing illegally in our country. All the patients had had a history of hematuria from a pediatric age and for this reason they had been submitted to the attention of urologists or nephrologists by the volunteer associations. At the time of diagnosis the mean eosinophilia was 1516 cells/mm^3^. In the computerized database of our operating unit, in five patients, vegetations in the bladder were shown by ultrasonography (Figure 1).

According to case definition, 4 were confirmed cases (1 had the detection of eggs in stool samples and 3 an histopathological diagnosis); 3 were probable cases (positivity of serology) and the remaining 2 patients were suspect cases (presence of swellings bladder and response to therapy, one each). All but 2 patients received a successful treatment with Praziquantel (40 mg/Kg per os in single dose) with a complete resolution of hematuria, whereas in the remaining two cases a second dose of drug was administered after about a month.

In the database, no urinary schistosomiasis case was reported until 2014. Eosinophilia and coming from Mali and Senegal, the two countries of origin of all seven Schistosomiasis cases, were the only significantly more frequent data in the population observed in the 2014–2016 period. However, there were no statistically significant differences with regard to the origin countries of patients who were attending our facility. About 50% of patients came from high or moderate endemic Schistosomiasis countries before and after 2014 (Table 2).

## 4. Discussion

Urinary schistosomiasis is a common problem in developing countries, while in our geographical area only a few cases have been observed. However, both the sporadic cases and the small outbreaks reported in European and the Middle East countries might be predictive of a further increase of cases in Europe due to migratory flows.

Herein, we report nine cases of urinary schistosomiasis diagnosed in the space of a few months between 2014 and 2016 at our outpatient clinic. Although the outpatient clinic was established in 2006, no cases of schistosomiasis were reported in the Apulia Region before 2014.

Our results confirm previous data of schistosomiasis in Northern Italy and in the metropolitan area of Barcelona. Moreover, although we considered a long period time, our cases only refer to migrants recently landed in Southern Italy, thereby resembling those of Beltrame et al. [14].

In general, we found a lower prevalence of schistosomiasis compared to that reported by Beltrame et al.; however, our data highlight a large circulation of* S. haematobium* among subjects from Mali. Coming Mali may have influenced the difference in prevalence between 2006 and 2016 in our outpatient clinic for different reasons. First, the increase parallels the augmented number of immigrants; in fact, in Italy an emergency-intensity level of immigration, which is still in progress, began in 2014, when the number of immigrants landing on the Italian shores quadrupled with respect to 2013 (i.e., 42,925 immigrants in 2013 versus 170,100 in 2014, according to the Reports of the Italian Ministry of the Interior) [16]. The epidemiology of immigration has also changed; indeed, all the seven patients in this report were from Mali and Senegal. Between 2013 and 2014 the number of migrants from these countries increased by 632% and 655%, respectively, from around 1,223 units to 7,735 (with regard to Malians) and from 472 to 3,094 (as regards the Senegalese). The same trend was also registered in 2015 and 2016. Therefore, Senegal and Mali are currently two of the top ten countries of origin of migrants in Italy [16].

According to the targets set by the WHO for schistosomiasis control, 92% of the African school-age population should carry out preventive treatment for schistosomiasis and/or geohelminthiasis. However, the percentage of children treated is just over 50% in Senegal and less than 10% in Mali (where during 2013 the economic and political situation rapidly worsened) [3].

On the other hand, the lack of reported cases up to 2015 in either Italy or Europe demonstrates a poor capacity of general practitioners and specialists (including infectious diseases specialists) in diagnosing schistosomiasis even in the presence of pathognomonic signs and symptoms (e.g., prolonged duration of hematuria along with eosinophilia and vegetations bladder).

Before coming to the observation of a specialist in infectious diseases, patients were evaluated by gastroenterologists, radiologists, urologists, and nephrologists. One patient admitted to the emergency department for hematuria was diagnosed with hemorrhagic cystitis and prescribed antibiotics. In three patients who underwent a bladder biopsy, ultrasound was already indicative of schistosomiasis. Therefore, the term “neglected disease” for schistosomiasis is still unquestionable [17].

Due to the increase in migratory pressure from high-endemic areas to Europe, one can expect an increase in the number of imported cases not necessarily admitted to infectious diseases clinic (as has been shown in our experience) and therefore misdiagnosed.

It is noteworthy that in our experience all the patients, including those not regularly present in Italy at the time of diagnosis, had ties to institutions and organizations supporting them to adjust to their new social environment. Therefore, an underestimation of cases of schistosomiasis in Apulia is somewhat plausible.

A careful and rigorous validation of diagnostic procedures would be needed for their adoption in the context of national public health programs aimed at the control and elimination of schistosomiasis. Analogous to screening for tuberculosis, it would be sensible to subject new arrivals to blood count and urinalysis in order to identify subjects worthy of further investigation.

## Figures and Tables

**Table 1:** Personal and clinical characteristics of the nine patients. NA: not available; Pt.: patient; Hb: hemoglobin; RBC: red blood cells; WBC: white blood cells; US: ultrasonographic.

Pt.	Age	Sex	Country of origin	Hb	Eosinophils cell/mm^3^	IgE	Urine RBC/field	Urine WBC/field	Proteinuria mg/dl	Eggs in urine	Serology	Histology	US features	Treatment response
(1)	19	M	Senegal	14,4	449	722	>40	25	50	Negative	Negative	NA	Negative	Yes
(2)	23	M	Mali	14,7	1990	1780	>40	ass	0	Negative	Negative	Positive	Swelling	Yes
(3)	18	M	Mali	14	5411	4580	Layer	75	50	Positive	Negative	NA	Negative	Yes
(4)	42	M	Mali	14,2	329	468	No RBC	ass	0	NA	NA	Positive	Swelling	Yes
(5)	19	M	Senegal	15,4	2183	15600	Layer	ass	150	Negative	Positive	NA	Swelling	Yes
(6)	21	M	Mali	14	243	NA	Layer	ass	0	NA	NA	Positive	Swelling	Yes
(7)	18	M	Nigeria	15	765	1440	1-2	ass	5	Negative	Negative	NA	Swelling	Yes
(8)	17	M	Mali	12,4	874	7970	>40	500	30	Negative	Positive	NA	Negative	Yes
(9)	41	M	Eritrea	12	1151	2520	>40	75	0	Negative	Positive	NA	Negative	Yes

**Table 2:** Statistic analysis. SD: standard deviation.

	Total 2006–2016	2006–2013	2014–2016	*p value*
Immigrants	1762	1261	501	

Average age	28,14	28,47	27,3	0,04
SD (range)	±11,07 (14–76)	±10,9 (14–76)	±11,32 (15–71)	

Male	1159 (65,8%)	801 (63,5%)	358 (71,4%)	0,002

Origin countries with high prevalence of schistosomiasis (%)	896 (50,85%)	643 (50,9%)	253 (50,5%)	0,852

Malian and Senegalese patients	83 (4,71%)	43 (3,4%)	40 (7,98%)	<0,0001

Prevalence of eosinophilia	209/1762 (11,8%)	126/1261 (9,9%)	84/501 (16,7%)	<0,0001

Average age of patients with eosinophilia SD Range	26,27 10,2 14–74	27,26 10,53 14–74	24,74 SD ± 9,55 15–52	0,08

Patients male with eosinophilia %	154/209 (73,7%)	87/126 (69%)	67/83 (80,72%)	0,08

## References

[1] Bennett J. E., Dolin R., Blaser M. J. (2015). *Mandell, Douglas, and Bennett's Principles and Practice of Infectious Diseases*.

[2] World Health Organization (2011). *Schistosomiasis: number of people treated in 2011*.

[3] World Health Organization (2013). *Schistosomiasis: progress report 2001–2011 and strategic plan 2012–2020*.

[4] Pearce E. J., MacDonald A. S. (2002). The immunobiology of schistosomiasis. *Nature Reviews Immunology*.

[5] Gryseels B., Polman K., Clerinx J., Kestens L. (2006). Human schistosomiasis. *The Lancet*.

[6] Colley D. G., Bustinduy A. L., Secor W. E. (2014). Human schistosomiasis. *The Lancet*.

[7] World Health Organization, 2017, http://www.who.int/schistosomiasis/Schistosomiasis_2012-01.png,?ua=1

[8] World Health Organization, http://gamapserver.who.int/mapLibrary/Files/Maps/Global_ShistoPrevalence_ITHRiskMap.png?ua=1

[9] http://www.cdc.gov/parasites/schistosomiasis/, 2016

[10] Roure S., Valerio L., Pérez-Quílez O. (2017). Epidemiological, clinical, diagnostic and economic features of an immigrant population of chronic schistosomiasis sufferers with long-term residence in a non-endemic country (North Metropolitan area of Barcelona, 2002-2016). *PLoS ONE*.

[11] http://ec.europa.eu/eurostat/data/database, 2016

[12] Caritas Italiana, Fondazione Migrantes. XXIII Rapporto immigrazione 2013. Tau Edition, 2014.

[13] Lingscheid T., Kurth F., Clerinx J. (2017). Schistosomiasis in European travelers and migrants: analysis of 14 years tropnet surveillance data. *Am J Trop Med Hyg*.

[14] Beltrame A., Buonfrate D., Gobbi F. (2017). The hidden epidemic of schistosomiasis in recent African immigrants and asylum seekers to Italy. *European Journal of Epidemiology*.

[15] Martelli G., Di Girolamo C., Zammarchi L. (2017). Seroprevalence of five neglected parasitic diseases among immigrants accessing five infectious and tropical diseases units in Italy: a cross-sectional study. *Clinical Microbiology and Infection*.

[16] http://ucs.interno.gov.it/ucs/contenuti/175723.htm (last visited on 15th May 2016)

[17] Danso-Appiah T. (2016). Schistosomiasis. *Neglected Tropical Diseases - Sub-Saharan Africa*.

